# Hybrid Multi-Objective Chameleon Optimization Algorithm Based on Multi-Strategy Fusion and Its Applications

**DOI:** 10.3390/biomimetics9100583

**Published:** 2024-09-25

**Authors:** Yaodan Chen, Li Cao, Yinggao Yue

**Affiliations:** School of Intelligent Manufacturing and Electronic Engineering, Wenzhou University of Technology, Wenzhou 325035, China

**Keywords:** chameleon optimization algorithm, logistic mapping, spiral oscillation, Lévy flight, refraction reverse learning, drones, path planning

## Abstract

Aiming at the problems of chameleon swarm algorithm (CSA), such as slow convergence speed, poor robustness, and ease of falling into the local optimum, a multi-strategy improved chameleon optimization algorithm (ICSA) is herein proposed. Firstly, logistic mapping was introduced to initialize the chameleon population to improve the diversity of the initial population. Secondly, in the prey-search stage, the sub-population spiral search strategy was introduced to improve the global search ability and optimization accuracy of the algorithm. Then, considering the blindness of chameleon’s eye turning to find prey, the Lévy flight strategy with cosine adaptive weight was combined with greed strategy to enhance the guidance of random exploration in the eyes’ rotation stage. Finally, a nonlinear varying weight was introduced to update the chameleon position in the prey-capture stage, and the refraction reverse-learning strategy was used to improve the population activity in the later stage so as to improve the ability of the algorithm to jump out of the local optimum. Eighteen functions in the CEC2005 benchmark test set were selected as an experimental test set, and the performance of ICSA was tested and compared with five other swarm intelligent optimization algorithms. The analysis of the experimental results of 30 independent runs showed that ICSA has stronger convergence performance and optimization ability. Finally, ICSA was applied to the UAV path-planning problem. The simulation results showed that compared with other algorithms, the paths generated by ICSA in different terrain scenarios are shorter and more stable.

## 1. Introduction

In recent years, with the increasing scale and dimension of engineering optimization problems, it is difficult for traditional optimization algorithms to give the optimal solution [1,2]. High-dimensional function optimization problems have important application value in theoretical research and practical production processes. Many engineering problems require finding optimal solutions under various constraints, such as research on logistics distribution path optimization, high-speed rail dynamic scheduling method problems, and multi-class twin support vector machine parameter optimization problems [3,4,5]. After they are mathematically modeled, they are essentially function optimization problems. High-dimensional complex function optimization usually refers to function optimization problems with dimensions exceeding 100 dimensions [6,7]. As the dimensions of optimization problems increase, the search space and the number of local optima grow exponentially along with the computational complexity, making these problems increasingly difficult to solve [8,9,10]. The swarm intelligence optimization algorithm, inspired by nature, is widely used in solving complex optimization problems such as workshop scheduling and path planning because of its simple principle, easy implementation, and strong global search ability [11,12,13]. In industrial production, process parameters have a crucial impact on product quality, production quality, and production efficiency. Swarm intelligence optimization algorithms can establish a relationship model between parameters and production results by analyzing a large amount of production data in order to find the optimal combination of process parameters. Swarm intelligence optimization algorithms can analyze the historical operating data of devices and explore potential patterns between device operating status and faults and thus achieve the prediction of device faults. Swarm intelligence optimization algorithms can optimize production scheduling and resource allocation based on factors such as production tasks, equipment conditions, and personnel arrangements. With the deepening of research, swarm intelligence optimization algorithms are constantly emerging, such as the typical particle swarm optimization algorithm (PSO), ant colony optimization algorithm (ACO), dung beetle optimization algorithm (DBO), gorilla troops optimization algorithm (GTO), butterfly optimization algorithm (BOA), and so on [14,15].

The chameleon swarm algorithm (CSA) [16] is a swarm intelligence optimization algorithm proposed by Malik in 2021. It was inspired by the effective hunting behavior of chameleons in deserts and forests. Chameleons are a unique and highly specialized evolutionary branch with a wide range of species and are known for their ability to change color to blend in with their surroundings. The chameleon has excellent eyesight, and its eyes can independently rotate 360 degrees [17]. In the process of predation, its sticky tongue chases food and forms a small suction cup, which can catch food at a speed of 2590 m/s [18]. The main difference between the CSA algorithm and other biological behavior algorithms is that chameleons’ eyes can rotate 360°, which increases the probability of finding prey, and they can use their high-speed, sticky tongue to catch prey [19]. The prey-capture efficiency is very high, so the solution effect is better.

At present, many scholars have studied the CSA algorithm and applied it in different fields, such as economic load distribution optimization of power system, energy saving optimization of wireless network, energy optimization management of microgrids, path planning, etc. [20]. These studies have checked the flexibility and reliability of the algorithm to some extent.

The remainder of this paper is organized as follows: Section 2 reviews the related work on the CSA algorithm. Section 3 details the standard CSA algorithm. Section 4 introduces the proposed multi-strategy improved chameleon optimization algorithm (ICSA). Section 5 presents the experimental results and analysis of this algorithm on the CEC2005 test set. Section 6 validates the effectiveness of the ICSA algorithm in UAV 3D path planning. Finally, Section 7 concludes the study.

## 2. Related Work

Similar to other swarm intelligence optimization algorithms, the CSA is prone to falling into the local optimum and has weak global search ability. In order to enhance the optimization ability of CSA, many scholars have made many improvements to the CSA.

Anitha et al. proposed a modified gray wolf-based chameleon swarm (MGW-CS) algorithm, which enhances the global search capability of the CSA by introducing the leadership hierarchy and predation strategy of grey wolf optimization [21]. Braik et al. proposed an enhanced chameleon swarm algorithm (ECSA) by integrating roulette wheel selection, Lévy flight methods, and an add-on position updating strategy to develop a further balance between exploration and exploitation conducts [22]. Salawudeen, Ahmed et al. designed two novel variants of CSA called quasi-oppositional CSA (QCSA) and quasi-Lévy-oppositional CSA (QLCSA). In QCSA, a quasi-oppositional-based learning operator is used to improve the diversification and intensification of CSA. The QLCSA incorporates the Lévy flight operator into the QCSA to avoid stagnation and local minimal entrapment [23]. Hu, Gang et al. proposed an enhanced hybrid CSA called CCECSA. It introduces the crisscross optimization algorithm to avoid premature convergence. It incorporates an elite guidance mechanism to accelerate the convergence, adopts a competitive substitution mechanism to replace the worst individual, and implements an interference strategy to prevent the algorithm from getting trapped in local optima [24].

The aforementioned improved algorithms enhance the optimization capability of the standard CSA to varying degrees, yet issues such as premature convergence and susceptibility to local optima persist in some complex multimodal problems. To further enhance the performance of the CSA, this paper proposes a multi-strategy improved chameleon optimization algorithm (ICSA), with improvements in the following four aspects: (1) initializing the chameleon population using logistic mapping; (2) introducing a sub-population spiral search strategy during the prey-search phase; (3) updating chameleon positions during eyes’ rotation with a combination of the Lévy flight strategy with cosine adaptive factor and a greedy selection strategy; and (4) introducing nonlinear varying weight and a refraction reverse-learning strategy to stimulate the activity of the chameleon population during the prey-capture phase. Finally, the ICSA is applied to 3D UAV path planning, verifying the effectiveness of the algorithm in solving complex optimization problems.

## 3. Standard Chameleon Swarm Algorithm

In the standard CSA, chameleon food hunting is mainly divided into three main stages: searching for prey, discovering prey through eyes’ rotation, and hunting prey [25,26].

### 3.1. Search for Prey

Like other creatures in nature, chameleons continuously adjust their positions during roaming prey searches, using previous positions and social experiences to track and discover prey [27]. The chameleons’ movement behavior during searching can be characterized based on the updating strategy of position, as in Equation (1):

(1) $$x_{t+1}^{i,j}=\left\{\begin{array}{l} x_{t}^{i,j}+p_{1}r_{2}\left(P_{t}^{i,j}-G_{t}^{j}\right)+p_{2}r_{1}\left(G_{t}^{j}-x_{t}^{i,j}\right)\;\;r\geq P_{p}\\ x_{t}^{i,j}+\mu \left(r_{3}\left(ub-lb\right)+lb\right)\mathrm{sgn}\left(rand-0.5\right)\;\;\;r<P_{p} \end{array}\right.$$

where $x_{t}^{i,j}$ presents the current position of the *i*-th chameleon in the *j*-th dimension at iteration *t*. $x_{t+1}^{i,j}$ is the new position of chameleon. $P_{t}^{i,j}$ is the individual best position of the *i*-th chameleon in the *j*-th dimension at iteration loop *t*. $G_{t}^{j}$ is the global best position of a chameleon by any chameleon in the *t*-th iteration.

*P_p_* represents the probability of perceiving prey. *p*_1_, *p*_2_ are positive coefficients controlling the algorithm’s development capability, and *r*_1_, *r*_2_, *r*_3_, *r*, and *rand* are random numbers generated uniformly in the range of [0, 1] [28]. sgn(*rand* − 0.5) indicates the chameleon’s rotation direction, taking a value of +1 or −1. If *r* ≥ *P_p_*, the chameleon can alter its position based on the prey observed in the search space [29]. Similarly, when *r* < *P_p_*, the chameleon will randomly explore the search space in various directions and regions to locate prey, increasing the likelihood of detecting nearby target prey. *μ* is a function of iterations parameter that decreases with the number of iteration, as in Equation (2) [30]:

(2) $$\mu =e^{{\left(-kt/T_{max}\right)}^{3}}$$

The original study [16] also performed a sensitivity analysis of the algorithm’s parameters, confirming that the algorithm exhibits optimal search performance when *k* = 3.5. Here, *T_max_* represents the maximum number of iterations, and *t* denotes the current iteration number.

### 3.2. Chameleon Eyes’ Rotation

Chameleons have a unique advantage over other organisms: the ability to independently rotate their eyes 360° to precisely locate prey. This process can be outlined in four steps [31]:

- The original position of the chameleon is the center of gravity (i.e., the origin);
- Discover the rotation matrix that identifies the position of prey;
- Update the chameleon’s position using the matrix of rotations at the center of gravity;
- Translate the chameleons back to the original position.

The aforementioned steps can be represented by the following equations [32]:

(3) $$x_{t+1}^{i}=m\left(x_{t}^{i}-{\bar{x}}_{t}^{i}\right)+{\bar{x}}_{t}^{i}$$

where $x_{t}^{i}$ presents the current position of the *i*-th chameleon at iteration *t*. ${\bar{x}}_{t}^{i}$ represents the center of the current position of the chameleon before rotation, which is also the average location of the individual in each dimension before position rotation. $x_{t+1}^{i}$ represents the new position of the *i*-th chameleon after rotation. *m* is a rotation matrix, so $m\left(x_{t}^{i}-{\bar{x}}_{t}^{i}\right)$ represents the rotating centered coordinates of the chameleon in the search space. *m* is defined as shown below [33]:

(4) $$m=R\left(\theta ,V_{z_{1},z_{2}}\right)$$

where *z*_1_ and *z*_2_ are two orthogonal vectors in an *n*-dimensional search space where the size of each vector is *d*. *R* denotes the rotation matrix on each axis [34].

(5) $$\theta =r\cdot \mathrm{sgn}\left(rand-0.5\right)\times \pi$$

where *θ* denotes the random rotation angle of the chameleon’s eyes. *r* is a random number in the range of [0, 1], and sgn(*rand* − 0.5) is either 1 or −1. Thus, the *θ* range is [−π, π].

### 3.3. Hunting Prey

The chameleon uses its tongue to attack prey. Once the tongue makes contact with the prey, it rapidly adheres to the surface of the prey, creating a small suction cup-like effect for effective capture [35]. Its position is updated slightly, as it can drop its tongue to double its length. The speed of the chameleon’s tongue when it is extended toward prey can be mathematically modeled according to Equation (6) [36]:

(6) $$\left\{\begin{array}{l} v_{t+1}^{i,j}=\lambda_{t}v_{t}^{i,j}+c_{1}r_{1}\left(G_{t}^{j}-x_{t}^{i,j}\right)+c_{2}r_{2}\left(P_{t}^{i,j}-x_{t}^{i,j}\right)\\ \lambda_{t}={\left(1-t/T_{\max}\right)}^{\rho \sqrt{t/T_{\max}}} \end{array}\right.$$

where $v_{t}^{i,j}$ presents the current velocity of the *i*-th chameleon in the *j*-th dimension at iteration *t*, and $v_{t+1}^{i,j}$ is new velocity of chameleon. *r*_1_ and *r*_2_ are random numbers in the range [0, 1]. *c*_1_ and *c*_2_ are two positive constants that control the influence of $G_{t}^{j}$ and $P_{t}^{i,j}$ on the chameleon’s speed. $\lambda_{t}$ is the inertia weight that is linearly decreased with the iterative generations, and *ρ* is equal to 1 [37].

Equation (7) was proposed to update the position of the chameleon:

(7) $$x_{t+1}^{i,j}=x_{t}^{i,j}+\frac{{\left(v_{t}^{i,j}\right)}^{2}-{\left(v_{t-1}^{i,j}\right)}^{2}}{2a}$$

where $v_{t}^{i,j}$ represents the current speed, $v_{t-1}^{i,j}$ denotes the speed from the previous iteration, and $x_{t}^{i,j}$ indicates the current position of the chameleon. *a* represents acceleration rate of the chameleon’s tongue projection, and it is defined as given in Equation (8):

(8) $$a=2590\times \left[1-e^{-\log\left(t\right)}\right]$$

where *t* denotes the current iteration number.

## 4. Multi-Strategy of the Chameleon Optimization Algorithm

### 4.1. Logistic Chaotic Map Initialization

The population initialization method has certain influence on the optimization effect and robustness of swarm intelligence algorithm. In the CSA algorithm, the initial position of chameleon swarm is randomly distributed, which may lead to uneven distribution or excessive concentration of the population and then lead to premature convergence of the algorithm falling into the local optimum or the convergence speed slowing down, which affects the final optimization effect and reduces the robustness of the algorithm [38].

Therefore, introducing appropriate chaotic sequence initialization can enrich the diversity of the initial population and reduce its above-mentioned correlation possibilities. At present, commonly used chaos optimization methods include logistic mapping, Singer mapping, Bernoulli mapping, Gauss mapping, etc. Among them, logistic mapping features a heterogeneous distribution, which helps prevent the initial population from being either too tightly clustered or too dispersed. Therefore, this paper selects logistic mapping to initialize the population, and the specific operation is shown in Equation (9):

(9) $$x^{i}=lb_{j}+L_{j}\times \left(ub_{j}-lb_{j}\right)\;\left(i=1,2,\ldots ,N,j=1,2,\ldots ,dim\right)$$

where *x^i^* is the initial position of the *i*-th chameleon among *N* chameleon individuals. *ub_j_* and *lb_j_* are the lower and upper bounds of the search space, respectively. *L_j_* is the generated logistic chaotic sequence. The definition of the logistic mapping is shown in Equation (10):

(10) $$L_{j+1}=rL_{j}\left(1-L_{j}\right)$$

where $L_{j}\in \left(0,1\right)$. *r* is the control parameter, and *r* is set to 0.3 in this paper.

### 4.2. Sub-Population Spiral Search Strategy

In the standard CSA, during the prey-search phase, chameleons have a 90% probability of moving towards the individual best position and the global best position. This local search method, however, is prone to getting trapped in the local optimum. Chameleons have a 10% probability of randomly exploring different directions and areas within the search space. This is a global search method, but it lacks guidance.

In order to balance the local and global search ability of the population at this stage, this paper firstly divides chameleon individuals into class A and class B according to their fitness values. Class A is chameleon individuals whose fitness value is lower than the average fitness value of the population, and class B is chameleon individuals whose fitness value is higher than the average fitness value of the population.

For a class A chameleon, the position is updated by using the above formula in Equation (11) in order to guide it to quickly search for prey by the individual best position and the global best position and speed up the convergence of the algorithm. For a class B chameleon, a new position update method is proposed. That is to say, on the basis of retaining a certain search randomness, guided by the global best position and combined with the variable spiral search strategy, more local search paths are developed for class B chameleons, thus improving the ability of the algorithm to jump out of the local optimum and find the global optimum. In summary, the position update formula of a chameleon in the prey-search stage is shown in Equation (11):

(11) $$x_{t+1}^{i,j}=\left\{\begin{array}{l} x_{t}^{i,j}+p_{1}r_{2}\left(P_{t}^{i,j}-G_{t}^{j}\right)+p_{2}r_{1}\left(G_{t}^{j}-x_{t}^{i,j}\right),\;\begin{array}{c} fit_{t}^{i}<meanfit_{t} \end{array}\\ x_{t+1}^{i,j}=G_{t}^{j}+e^{zl}\cdot \cos\left(2\pi l\right)\cdot \left(G_{t}^{j}-x_{t}^{i,j}\right)\cdot \mathrm{sgn}\left(\mathrm{rand}-0.5\right),\;\begin{array}{c} fit_{t}^{i}\geq meanfit_{t} \end{array}\\ z=e^{k\cdot \cos\left(\pi \cdot \left(1-\left(t/T\right)\right)\right)} \end{array}\right.$$

where $fit_{t}^{i}$ is the fitness value of *i*-th chameleon at iteration *t*, *meanfit_t_* is the average fitness value of the population at iteration *t*, *l* is the uniform random number of [−1, 1], and *k* is the variation coefficient, which is set to 5 in this paper.

### 4.3. Lévy Flight Strategy with Cosine Adaptive Factor and Greedy Selection Strategy

#### 4.3.1. Lévy Flight Strategy with Cosine Adaptive Factor

In the stage of chameleons’ eyes turning to find prey, the chameleon changes the search direction and search area to approach the center of gravity with the help of the current-position center of gravity and rotation matrix [39]. While this process can enhance the global search capability of the chameleon algorithm, it introduces a high degree of randomness. This randomness may cause the algorithm to inadequately cover the search space during early iterations, potentially missing the optimal solution. Additionally, it may lack adaptability in later stages, resulting in suboptimal search performance [40].

**Figure 1:**
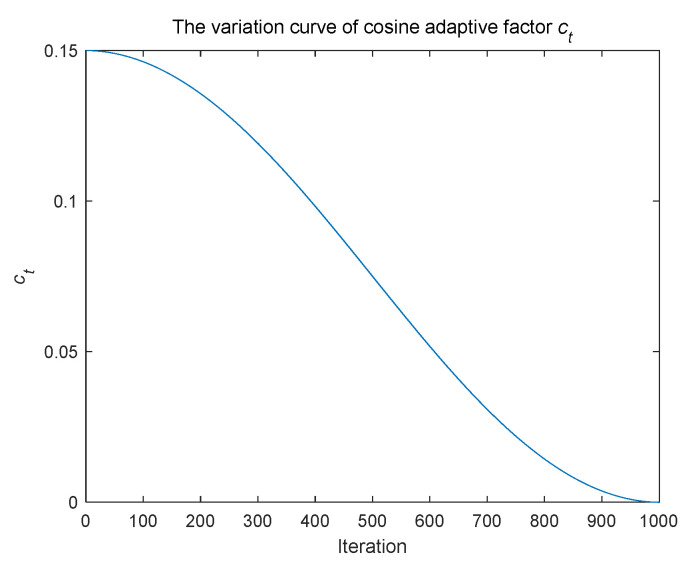
The variation curve of cosine adaptive factor *c_t_*.

To solve this problem, this paper introduces the Lévy flight strategy with cosine adaptive factor. The formula for calculating the cosine adaptive factor is as follows:

(12) $$c_{t}=0.075\left(1+\cos\left(\frac{\pi t}{T_{\max}}\right)\right)$$

where *t* denotes the current iteration number. *T*_max_ represents the maximum number of iterations. The variation curve of cosine adaptive factor *c_t_* with the number of iterations is shown in Figure 1.

Lévy flight is a random flight with alternating near and far search ranges, and the probability distribution of its step size is heavy-tailed distribution. When defining walking in a space larger than one dimension, the walking step size is a random direction of each peer. The expression for the Lévy step length is as follows:

(13) $$L\mathrm{evy}\left(\beta \right)=\frac{u}{{\left|v\right|}^{\frac{1}{\beta }}}$$

where $u\;\sim \;N\left(0,\sigma_{u}^{2}\right)$, $v\;\sim \;N\left(0,\sigma_{v}^{2}\right)$. $\sigma_{u}$, $\sigma_{v}$ are defined as follows:

(14) $$\left\{\begin{array}{l} \sigma_{u}=\frac{\Gamma \left(1+\beta \right)\sin\left(\pi \beta /2\right)}{2^{\left(\beta -1\right)/2}\Gamma \left[\left(1+\beta \right)/2\right]\beta }\\ \sigma_{v}=1 \end{array}\right.$$

where Γ represents the standard gamma function. *β* is a constant in the range of [0, 2], and it is set to 1.5 in this paper.

In summary, after updating its position through eye rotation, the chameleon uses the Lévy flight strategy with cosine adaptive factor to expand its search area. As shown in Figure 1, the cosine adaptive factor *c_t_* stabilizes at a higher value during early iterations, increasing the probability of long-step movements, which helps expand the global search range. In later iterations, *c_t_* stabilizes at a lower value, increasing the probability of short-step movements, which allows for more precise local searches and enhances optimization accuracy. Furthermore, considering the guiding influence of the group’s optimal position, the position update formula after incorporating the Lévy flight strategy with cosine adaptive factor during the eye-rotation stage is presented in Equation (15):

(15) $$xl_{t}^{i,j}=x_{t}^{i,j}+c_{t}\cdot \left(G_{t}^{j}-x_{t}^{i,j}\right)\cdot L\mathrm{evy}{\left(\beta \right)}_{t}^{i,j}$$

#### 4.3.2. Greedy Selection Strategy

Due to the random characteristics of Lévy flight, the updated chameleon position does not necessarily have better fitness. Thus, the greedy strategy is adopted to choose whether to update the new position, and the formula is shown in Equation (16):

(16) $$x_{t}^{i,j}=\left\{\begin{array}{l} x_{t}^{i,j},\;\begin{array}{c} fitness\left(x_{t}^{i,j}\right)\geq fitness\left(xl_{t}^{i,j}\right) \end{array}\\ xl_{t}^{i,j},\;\begin{array}{c} fitness\left(x_{t}^{i,j}\right)<fitness\left(xl_{t}^{i,j}\right) \end{array} \end{array}\right.$$

where $fitness\left(x_{t}^{i,j}\right)$ and $fitness\left(xl_{t}^{i,j}\right)$ represent the fitness value of the chameleon’s eye-rotation position update and the fitness value calculated by the Lévy flight strategy update position with cosine adaptive factor, respectively.

### 4.4. Nonlinear Varying Weight and Refraction Reverse-Learning Strategy

#### 4.4.1. Nonlinear Varying Weight

**Figure 2:**
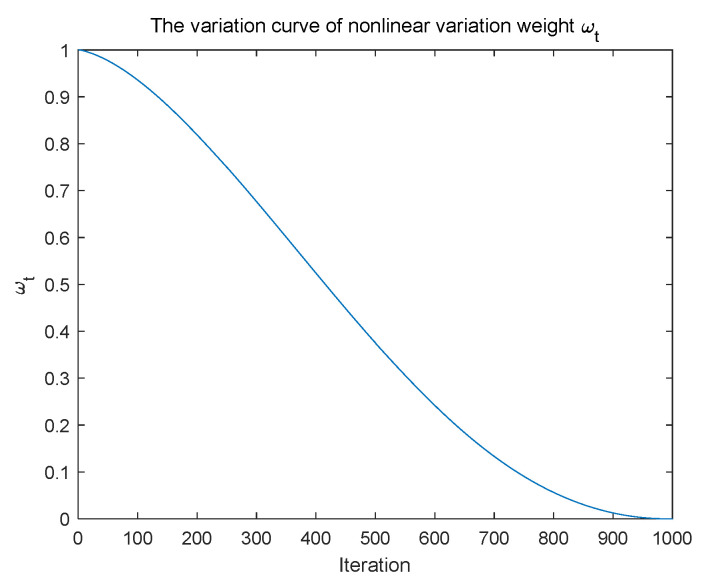
The variation curve of nonlinear varying weight *ω_t_*.

As seen in Equation (6), the speed update during the chameleon’s prey-hunting stage is similar to that of particle swarm optimization (PSO), and *c*_1_ and *c*_2_ are the control factors of individual optimal position and global optimal position for velocity update. In the early stage of algorithm iteration, the chameleon position is far away from the optimal position. In order to achieve faster convergence speed, it is expected to increase *c*_1_ and *c*_2_. In the later iteration stage of the algorithm, the chameleon position is relatively close to the optimal position. In order to ensure the convergence accuracy and avoid falling into the local optimum, it is expected to reduce *c*_1_ and *c*_2_. In this paper, a nonlinear varying weight is adopted, as shown in Equation (17):

(17) $$\omega_{t}={\left(1-\frac{t}{T_{\max}}\right)}^{2\sqrt{\frac{t}{T_{\max}}}}$$

where *t* denotes the current iteration number. *T*_max_ represents the maximum number of iterations. The variation curve of nonlinear varying weight *ω_t_* with the number of iterations is shown in Figure 2.

The speed update formula is changed as follows:

(18) $$v_{t+1}^{i,j}=\lambda_{t}v_{t}^{i,j}+\omega_{t}r_{1}\left(G_{t}^{j}-x_{t}^{i,j}\right)+\omega_{t}r_{2}\left(P_{t}^{i,j}-x_{t}^{i,j}\right)$$

#### 4.4.2. Refraction Reverse-Learning Strategy

**Figure 3:**
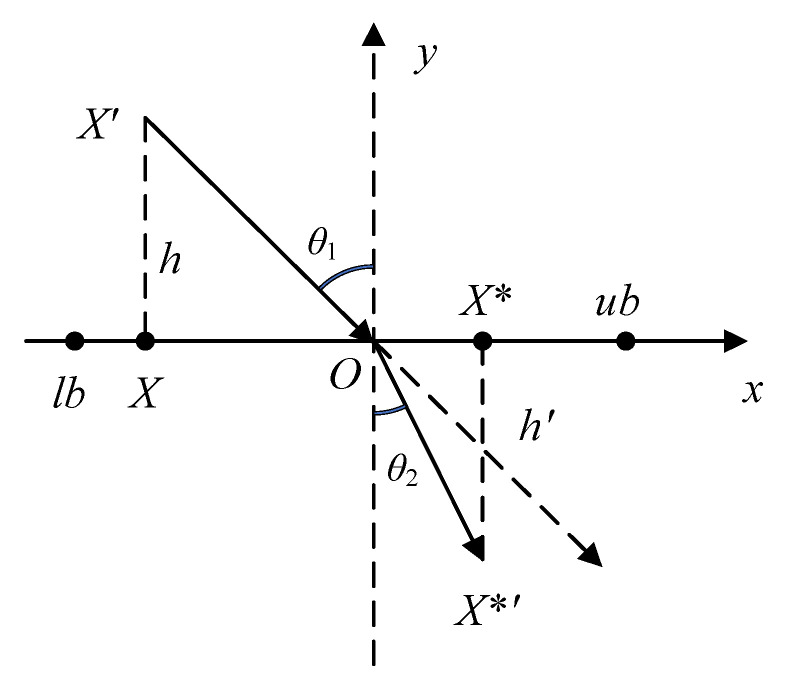
Schematic diagram of refraction reverse learning.

Similar to many swarm intelligence optimization algorithms, the CSA can easily fall into the local optimum in the later iteration stage because of its high population similarity and low activity. To address this issue, this paper introduces a refraction reverse-learning strategy following the chameleon’s prey-hunting position update. The refraction reverse-learning strategy is based on the refraction principle of light, and its principle is shown in Figure 3 below.

In Figure 3, *O* is the midpoint of *ub* and *lb*, which are the upper and lower limits of the interval, respectively. The refracted ray OX*′ is formed by the refraction of the incident ray *X*′*O* by boundary mass, and the heights are *h*′ and *h*, respectively.

Assuming that the refractive index of free space and dielectric space is the same, then *θ*_1_ = *θ*_2_. X* and *X*, respectively, represent the projections of OX*′ and *X*′*O* on the x-axis, respectively. At this time, the expression of the refraction imaging principle can be described as follows:

(19) $$\frac{\frac{lb+ub}{2}-X}{X^{*}-\frac{lb+ub}{2}}=\frac{h}{h^{\prime }}$$

Let $k=\frac{h}{h^{\prime }}$. *k* denotes the dynamic scaling factor, and its value is equal to *t*, which changes linearly with the current number of iterations; then, the expression of X* is as follows:

(20) $$X^{*}=\frac{lb+ub}{2}+\frac{lb+ub}{2t}-\frac{X}{t}$$

Through the refraction reverse-learning strategy, the search radius is dynamically adjusted. In the early stage of algorithm iteration, potential feasible solutions are developed on a large scale to increase the possibility of global search. In the later stage of algorithm iteration, high-quality solutions are developed in a small-scale and fine way to improve population activity, which will improve the optimization accuracy and convergence speed to a certain extent. However, the updated chameleon position does not necessarily have better fitness, so it is necessary to adopt greedy strategy according to Equation (16) to choose whether to update the new position.

### 4.5. Pseudo-Code of the Multi-Strategy Improved Chameleon Algorithm

To sum up, the multi-strategy improved chameleon algorithm (ICSA) (Algorithm 1) proposed in this paper can increase the group diversity to a certain extent, balance the local search and global search capabilities of the algorithm, and improve the optimization ability of the algorithm. The pseudo-code of the ICSA is as follows:
**Algorithm 1:** ICSA pseudo-codeBegin1.  Setting parameters: *N* ← population size, *dim* ← spatial dimension,         *T*_max_ ← maximum number of iterations,          *lb* ← lower limit of the search space,          *ub* ← upper limit of the search space. 2.  Initialize the population *X* by logistic chaotic mapping using Equation (9).3.  Calculate the individual fitness to determine the individual best position *P* and the global best position *G*.4.  While (*t ≤ T*_max_).5.  Calculate the mean individual fitness *meanfit_t._*6.  for *i* = 1 *to N*7.  Update the chameleon’s position based on Equation (11).8.  end for9.  Compute the cosine adaptive factor *c_t_* using Equation (12).10.for *i* = 1 *to N*11.Update the chameleon’s position according to Equations (3)–(5).12.Calculate the chameleon’s random position using the Lévy flight strategy with the  cosine adaptive factor using Equation (15).13.Determine whether to update the chameleon’s position to the random position based  on the greedy selection using Equation (16).14.end for15.Calculate the nonlinear varying weight *ω_t_* according to Equation (17).16.for *i* = 1 *to N*17.Update the chameleon’s velocity using Equation (18).18.Update the chameleon’s position using Equation (7).19.Calculate the chameleon’s expanded position by incorporating the refraction reverse  learning strategy as defined by Equation (20).20.Determine whether to update the chameleon’s position to the expanded position based  on the greedy selection using Equation (16).21.end for22.Calculate the individual fitness and update the individual best position *P_t_* and the global best position *G_t_*.23.*t* = *t* + 1.24.end while25.Output the fitness array, the global best position, and the optimal fitness value.End

### 4.6. Flowchart of the Multi-Strategy Improved Chameleon Algorithm

**Figure 4:**
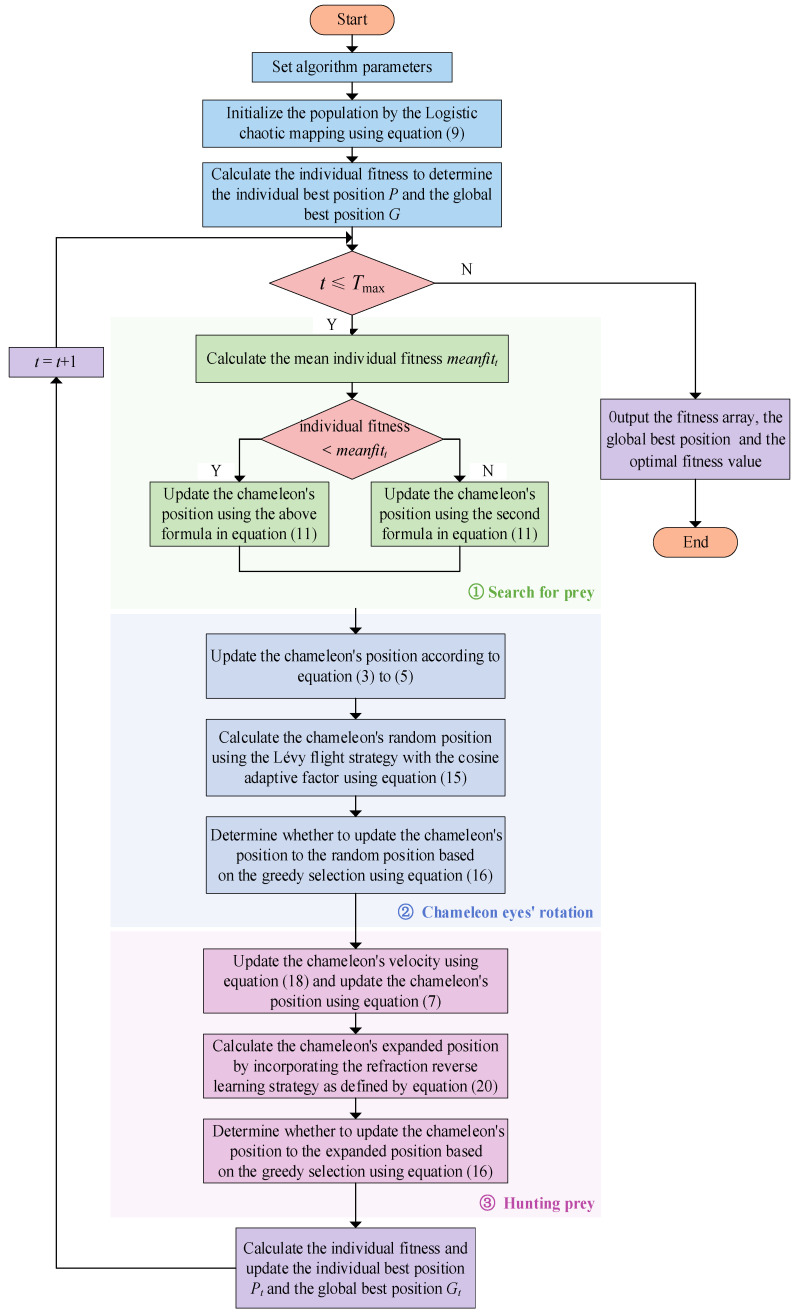
The flowchart of ICSA algorithm.

The flowchart of the multi-strategy improved chameleon algorithm (ICSA) is shown in Figure 4.

## 5. Experimental Results and Simulation Analysis

### 5.1. Experimental Environment Settings

To verify the performance of the ICSA algorithm, the experimental environment was configured as follows: The operating system was Windows 10 (64-bit), the processor was an Intel(R) Core(TM) i7-10875H with a main frequency of 2.3 GHz, the computer memory was 16 GB, and the simulation software used was MATLAB R2020b.

### 5.2. Comparison Algorithms and Parameter Settings

To validate the performance of the ICSA algorithm, we selected the classic CSA algorithm and four other swarm intelligence optimization algorithms that are either classic or have shown good performance in recent years for comparative analysis. These algorithms include the dung beetle optimizer (DBO) [41], the subtraction-average-based optimizer (SABO) [42], the gorilla troop optimization (GTO) [43], and the ant colony optimization (ACO) [44].

CEC2005 was chosen as the benchmark function. To ensure the reliability of the test results, the population size for all algorithms was set to 30 and the number of iterations to 1000. The specific parameter settings for each algorithm are detailed in Table 1.

### 5.3. Test Functions

To fairly evaluate the effectiveness of the ICSA algorithm, we selected 18 test functions from the CEC2005 test set for validation. These included six unimodal functions (*F*_1_(*x*)~*F*_6_(*x*)), five multimodal functions (*F*_7_(*x*)~*F*_11_(*x*)), and seven fixed-dimension functions (*F*_12_(*x*)~*F*_18_(*x*)). Unimodal functions were used to evaluate the algorithm’s local search capability, multimodal functions to evaluate its global search capability, and fixed-dimension functions to assess the balance between exploration and exploitation. Detailed information about the benchmark test functions is presented in Table 2.

### 5.4. Optimization Results and Analysis of Test Functions

Using the experimental environment and parameter settings described above, each algorithm was run independently 30 times on the 18 test functions. The results are presented in Table 3, including metrics such as average value, standard deviation, best value, and worst value.

To eliminate randomness, the average value of the experimental results was used to measure the optimization accuracy of the algorithms. By using the mean value as a comparison metric, it is evident that the ICSA algorithm achieves better solutions in the test functions compared to the standard CSA algorithm, indicating that the improvements enhance the algorithm’s optimization accuracy. Compared to other optimization algorithms, ICSA consistently finds the optimal or near-optimal solutions, demonstrating its strong optimization capability, with GTO being the next best performer.

The standard deviation of the experimental results measures the degree of deviation in the algorithm’s results and is a key indicator of algorithm stability. Using the standard deviation as a comparison metric, it is clear that ICSA exhibits stable and superior optimization performance compared to both the standard CSA algorithm and other optimization algorithms.

In summary, within the specified number of iterations, the optimization accuracy and robustness of ICSA show significant advantages.

### 5.5. Convergence Curve Analysis

**Figure 5:**
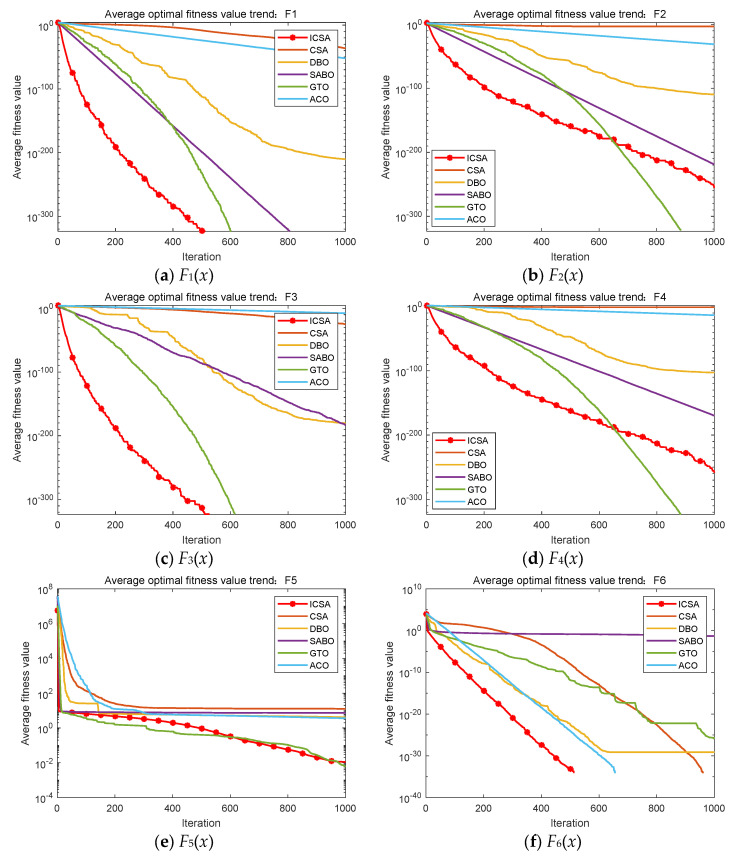

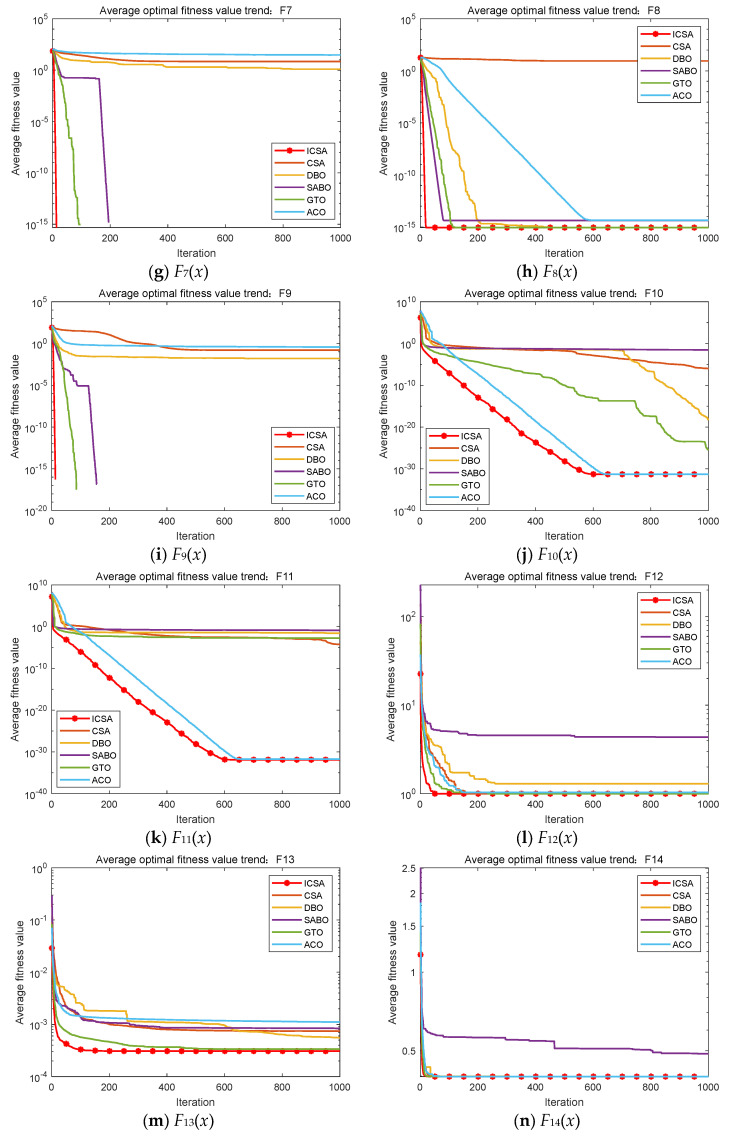

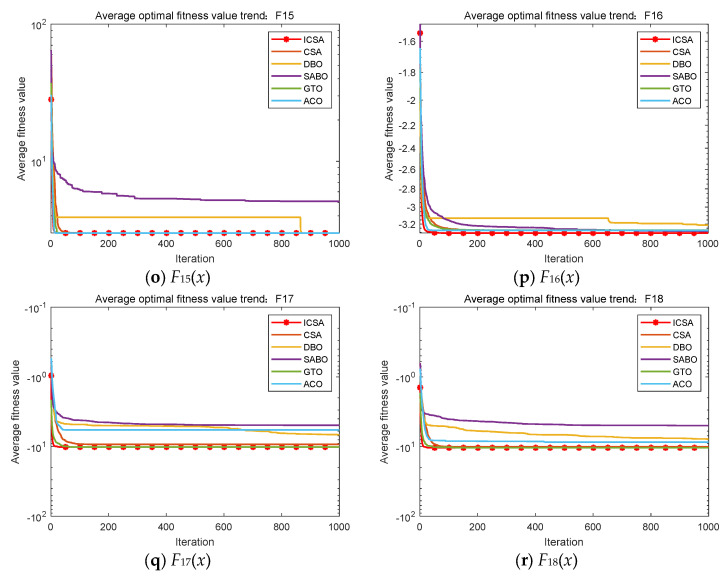
Convergence curves of test functions (*N* = 30).

The convergence curve visually demonstrates the convergence speed and optimization accuracy of the algorithms. Figure 5 shows the convergence curves for each algorithm obtained simultaneously in the aforementioned experiments. The horizontal axis of each curve represents the number of iterations, and the vertical axis represents the fitness value.

It is evident that, compared to the standard CSA algorithm, ICSA significantly accelerates convergence and enhances optimization accuracy. Compared to other optimization algorithms, ICSA also demonstrates substantial advantages in both convergence speed and optimization accuracy. In summary, within the specified number of iterations, ICSA exhibits significant advantages in convergence speed and optimization accuracy.

## 6. Application and Analysis of ICSA in UAV Path Planning

To validate the effectiveness of the ICSA algorithm in addressing real-world optimization problems, this paper applies ICSA to the UAV 3D path planning. The objective of UAV 3D path planning is to find a safe and efficient route from the starting position to the target position under specific constraints [45].

### 6.1. Terrain Obstacle Model

For the UAV path-planning problem, terrain obstacle modeling is the first step. This study selected a three-dimensional space of 1000 m × 1000 m × 100 m, used an exponential function to simulate mountain peaks as the terrain, and modeled obstacle threat areas using cylinders [46]. The mathematical model for the mountain peak terrain is as follows:

(21) $$z\left(x,y\right)=\sum_{i=1}^{n}h_{i}\exp\left[-{\left(\frac{x-x_{i}}{x_{si}}\right)}^{2}-{\left(\frac{y-y_{i}}{y_{si}}\right)}^{2}\right]$$

where *z* represents the mountain peak terrain data. *n* denotes the number of mountain peaks. *h_i_* is the height of the *i*-th mountain peak. [*x_i_*, *y_i_*] is the center coordinate of the *i*-th mountain peak, and *x_si_* and *y_si_* are the descending slopes of the *i*-th mountain peak along the *x*-axis and *y*-axis, respectively.

### 6.2. Cost Function

#### 6.2.1. Path Length Cost

Path length is a crucial factor in UAV path planning. A shorter path length indicates a shorter time required for the UAV to complete the mission, thereby reflecting higher path-planning quality [47]. After determining the starting and ending points of the path, *M* track points are selected between these points. Using the cubic spline interpolation method, the planned path is then discretized into a path with *N* nodes, where each node *X_i_* corresponds to a spatial coordinate (*x_i_*, *y_i_*, *z_i_*). The path length cost is the sum of the Euclidean distances between adjacent nodes in the path, calculated as follows:

(22) $$F_{1}\left(X\right)=\sum_{i=1}^{N-1}\sqrt{{\left(x_{i+1}-x_{i}\right)}^{2}+{\left(y_{i+1}-y_{i}\right)}^{2}+{\left(z_{i+1}-z_{i}\right)}^{2}}$$

#### 6.2.2. Flight Altitude Cost

Choosing the correct flight altitude is also critical for UAV path planning. A reasonable altitude can reduce collision risk and enhance safety while also affecting flight speed and energy consumption, thus influencing flight efficiency [48].

Ground obstacle threat areas are modeled as cylinders, with mountain peaks representing the terrain. Therefore, effective path planning should ensure the flight path avoids intersecting threat areas and that the flight altitude at any node is above the corresponding terrain height. If the flight path is valid, the flight altitude cost is calculated using the formula in the upper part of Equation (23). Otherwise, the flight altitude cost is set to 10^32^. The total flight altitude cost calculation is as follows:

(23) $$F_{2}\left(X\right)=\left\{\begin{array}{l} \sum_{n=1}^{N}\left|z_{n}-\bar{z}\right|,\mathrm{IsPathOk}=\mathrm{true}\\ 10^{32},\mathrm{IsPathOk}=\mathrm{false} \end{array}\right.$$

where $z_{n}$ represents the altitude of each path node above ground level, and $\bar{z}$ denotes the average altitude of the respective path nodes.

#### 6.2.3. Turning Angle Cost

The UAV’s turning angle directly affects its stability and maneuverability. During flight, the turning angle should not exceed the predefined maximum turning angle. The formula for angle change during UAV flight is as follows:

(24) $$\theta_{i}=\arccos\frac{\alpha_{i}\beta_{i}}{\left|\alpha_{i}\right|\left|\beta_{i}\right|}$$

where *α_i_* represents the difference in spatial coordinates between the path node *X*_*i*+1_ (*x*_*i*+1_, *y_i_*_+1_, *z_i_*_+1_) and the path node *X_i_* (*x_i_*, *y_i_*, *z_i_*). *β_i_* represents the difference in spatial coordinates between the path node *X_i_*_+2_ (*x_i_*_+2_, *y*_*i*+2_, *z_i_*_+2_) and the path node *X_i_*_+1_ (*x_i_*_+1_, *y_i_*_+1_, *z_i_*_+1_). The formula for calculating the turning angle cost is as follows:

(25) $$F_{3}\left(X\right)=\sum_{i=1}^{N-1}\left|\theta_{i+1}-\theta_{i}\right|$$

#### 6.2.4. Total Cost Function

By integrating the three path optimization-related cost functions discussed above, the total cost function is defined as the weighted sum of the following components:

(26) $$F\left(X\right)=b_{1}F_{1}\left(X\right)+b_{2}F_{2}\left(X\right)+b_{3}F_{3}\left(X\right)$$

The weights *b*_1_, *b*_2_, and *b*_3_ are coefficients that satisfy *b*_1_ + *b*_2_ + *b*_3_ = 1, and they can be adjusted according to different scenarios. Under the condition of safe obstacle avoidance, a smaller total cost function value indicates smoother and more efficient path planning. In this paper, the weights *b*_1_, *b*_2_, and *b*_3_ are set to 0.4, 0.4, and 0.2, respectively.

### 6.3. UAV Path-Planning Simulation

In this study, two different threat obstacle terrains were selected. Terrain 1 contains seven mountain peaks and one cylindrical obstacle threat area, while terrain 2 contains four mountain peaks and one cylindrical obstacle threat area. The specific parameter settings for the peaks and cylindrical obstacle threat areas are provided in Table 4 and Table 5.

**Figure 6:**
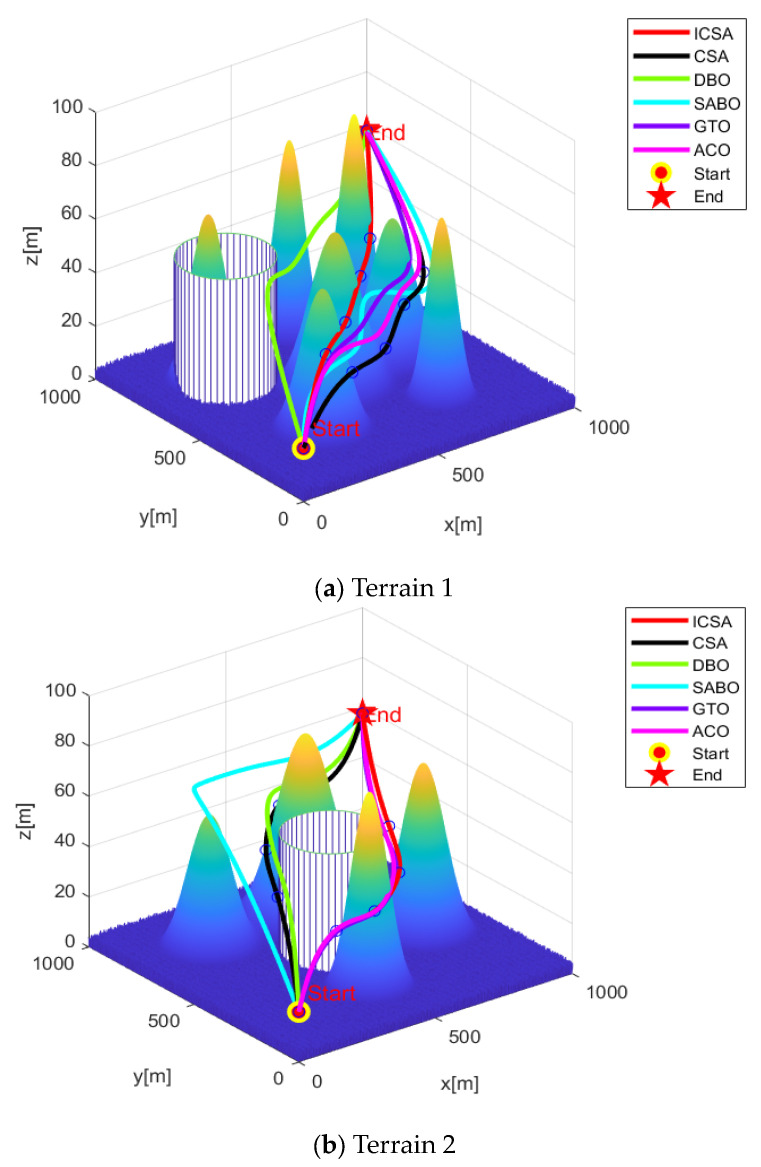
UAV 3D path planning (*N* = 100).

The previously proposed ICSA was applied alongside five other swarm intelligence optimization algorithms for path-planning simulations and comparative analysis in these terrains. Each algorithm was tested independently 20 times, with the population size set at 100 and the maximum number of iterations set at 1000. The starting coordinates were (0, 0, 20) and the ending coordinates were (977, 970, 60). The path-planning simulation results for the two different terrains are shown in Figure 6.

**Figure 7:**
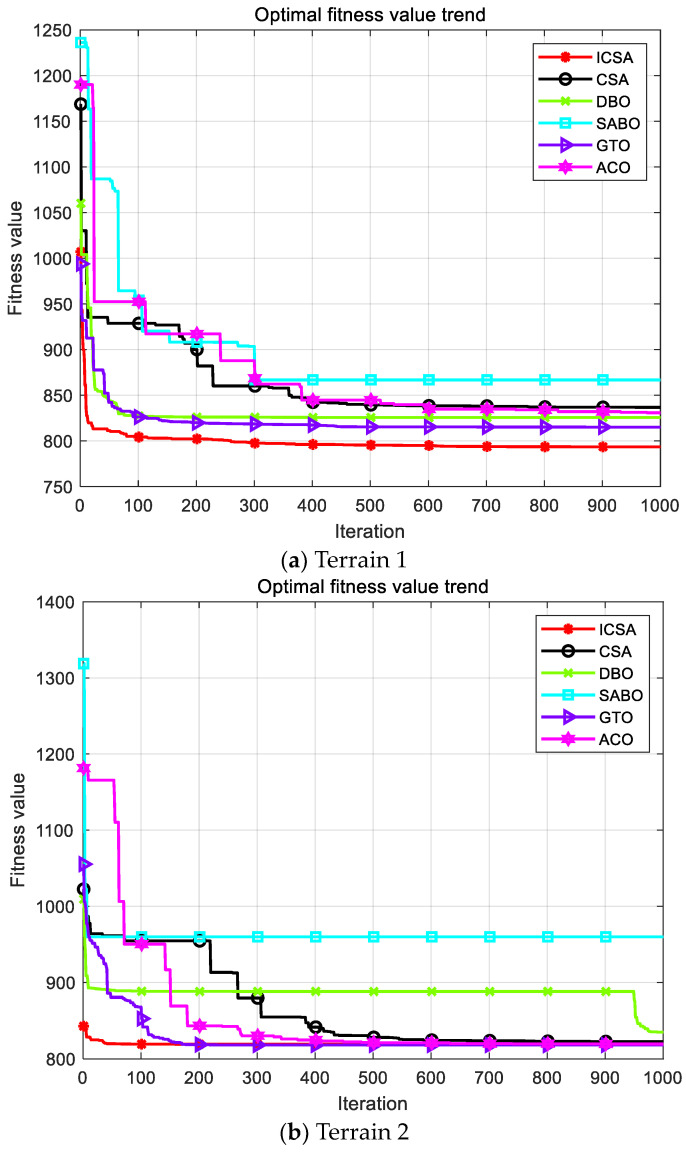
Total cost function convergence curve.

As shown in Figure 6, terrain 1 is more complex, while terrain 2 is relatively simpler. In both terrains, the ICSA algorithm demonstrates superior path planning compared to the standard CSA algorithm, resulting in shorter flight distances. Figure 7 indicates that the ICSA algorithm achieves a lower total cost, faster convergence speed, and better path-planning performance.

Table 6 provides a summary of the UAV path-planning results in the two different terrains, including metrics such as the average value, standard deviation, best value, and worst value.

Using the average value as a comparison metric, it is evident that the ICSA achieves a smaller total cost function value compared to the standard CSA algorithm, demonstrating that the improved strategy can effectively enhance the algorithm’s optimization accuracy. When compared to other optimization algorithms, the ICSA consistently finds the optimal solution or gets closer to it, indicating significant advantages, with the next best performance observed in the GTO algorithm.

The standard deviation of the experimental results primarily measures the deviation of the algorithm’s results, serving as a key indicator of algorithm stability. Using standard deviation as a comparison metric, the ICSA exhibits stable and excellent optimization performance relative to both the standard CSA algorithm and other optimization algorithms. Overall, the ICSA algorithm demonstrates superior performance in solving the 3D path-planning problem.

## 7. Conclusions

The standard CSA algorithm faces issues such as slow convergence speed, poor robustness, and a tendency to get trapped in local optima, indicating significant room for performance improvement in practical applications. Thus, this paper introduces a multi-strategy improved chameleon optimization algorithm (ICSA), incorporating strategies such as logistic mapping, sub-population spiral search strategy, Lévy flight with cosine adaptive factor, greedy strategy, and refraction reverse-learning strategy.

Simulation experiments using the CEC2005 standard test functions demonstrated that the ICSA algorithm offers faster convergence speed, higher optimization accuracy, and greater robustness compared to five other swarm intelligence optimization algorithms. Finally, the ICSA was applied to the UAV path-planning problem, accounting for path length cost, altitude cost, and turning angle cost. The ICSA shows superior performance in terms of path-planning length, convergence speed, and stability, effectively achieving 3D path planning across different terrains.

Like other intelligent optimization algorithms, ICSA has strong adaptability. It can also be verified through experiments that ICSA shows good performance in classic engineering problems such as pressure vessel design, three-bar truss design, and tension-compression spring design. Going forward, the performance of the ICSA algorithm could be further enhanced based on the current research. It is believed that ICSA will have certain application value in various industrial fields.

## Figures and Tables

**Table 1:** Algorithm parameter settings.

Algorithm	Main Parameter Configurations
ICSA	*ρ* = 1, *r* = 0.3, *β* = 1.5
CSA	*P_p_ =* 0.1, *k* = 3.5, *ρ* = 1
DBO	*B* = 0.3, *k* = 0.1, *S* = 0.5
SABO	-
GTO	*p* = 0.03, *β* = 3, *ω* = 0.8

**Table 2:** Test functions.

Function Type	Function Expression	Dimension	Search Range	Optimal Value
Unimodal Functions	$F_{1}\left(x\right)=\sum_{i=1}^{n}x_{i}^{2}$	30	[−100, 100]	0
	$F_{2}\left(x\right)=\sum_{i=1}^{n}\left|x_{i}\right|+\prod_{i=1}^{n}\left|x_{i}\right|$	30	[−10, 10]	0
	$F_{3}\left(x\right)={\sum_{i=1}^{n}\left(\sum_{j-1}^{i}x_{j}\right)}^{2}$	30	[−100, 100]	0
	$F_{4}\left(x\right)=\max_{i}\left\{\left|x_{i}\right|,1\leq i\leq n\right\}$	30	[−10, 10]	0
	$F_{5}\left(x\right)=\sum_{i=1}^{n-1}\left[100{\left(x_{i+1}-x_{i}^{2}\right)}^{2}+{\left(x_{i}-1\right)}^{2}\right]$	30	[−30, 30]	0
	$F_{6}\left(x\right)={\sum_{i=1}^{n}\left[x_{i}+0.5\right]}^{2}$	30	[−100, 100]	0
Multimodal Functions	$F_{7}\left(x\right)=\sum_{i=1}^{n}\left[x_{1}^{2}-10\cos\left(2\pi x_{i}\right)+10\right]$	30	[−5.12, 5.12]	0
	$\begin{array}{r} F_{8}\left(x\right)=-20\exp\left(-0.2\sqrt{\frac{1}{n}\sum_{i=1}^{n}x_{i}^{2}}\right)-\;\\ \exp\left(\frac{1}{n}\sum_{i=1}^{n}\cos\left(2\pi x_{i}\right)\right)+20+e \end{array}$	30	[−32, 32]	0
	$F_{9}\left(x\right)=\frac{1}{4000}\sum_{i=1}^{n}x_{i}^{2}-\prod_{i=1}^{n}\cos\left(\frac{x_{i}}{\sqrt{i}}\right)+1$	30	[−600, 600]	0
	$\begin{array}{l} F_{10}\left(x\right)=\frac{\pi }{n}\left\{10\sin\left(\pi y_{1}\right)+\right.\sum_{i=1}^{n-1}{\left(y_{i}-1\right)}^{2}\left[1+10\sin^{2}\left(\pi y_{i+1}\right)\right]\\ \;\;+\left.{\left(y_{n}-1\right)}^{2}\right\}+\sum_{i=1}^{n}u\left(x_{i},10,100,4\right) \end{array}$ $y_{i}=1+\frac{x_{i}+1}{4}$ $u\left(x_{i},a,k,m\right)=\left\{\begin{array}{l} k{\left(x_{i}-a\right)}^{m},x_{i}>a\\ 0,-a<x_{i}<a\\ k{\left(-x_{i}-a\right)}^{m},x_{i}<-a \end{array}\right.$	30	[−50, 50]	0
	$\begin{array}{r} F_{11}\left(x\right)=0.1\left\{\sin^{2}\left(3\pi x_{1}\right)+\right.\sum_{i=1}^{n}{\left(x_{i}-1\right)}^{2}\left[1+\sin^{2}\left(3\pi x_{i}+1\right)\right]\cdot \\ \left.{\left(x_{n}-1\right)}^{2}\left[1+\sin^{2}\left(2\pi x_{n}\right)\right]\right\}+\sum_{i=1}^{n}u\left(x_{i},5,100,4\right)\;\; \end{array}$	30	[−50, 50]	0
Fixed-Dimension Functions	$F_{12}\left(x\right)={\left(\frac{1}{500}+\sum_{j=1}^{25}\frac{1}{j+\sum_{i=1}^{2}{\left(x_{i}-a_{ij}\right)}^{6}}\right)}^{-1}$	2	[−65, 65]	1
	$F_{13}\left(x\right)=\sum_{i=1}^{11}{\left[a_{i}-\frac{x_{1}\left(b_{i}^{2}+b_{i}x_{2}\right)}{b_{i}^{2}+b_{i}x_{3}+x_{4}}\right]}^{2}$	4	[−5, 5]	0.0003
	$F_{14}\left(x\right)={\left(x_{2}-\frac{5.1}{4\pi^{2}}{x_{1}}^{2}+\frac{5}{\pi }x_{1}-6\right)}^{2}+10\left(1-\frac{1}{8\pi }\right)\cos x_{1}+10$	2	[−5, 5]	0.398
	$\begin{array}{r} F_{15}\left(x\right)=\left[1+{\left(x_{1}+x_{2}+1\right)}^{2}\left(19-14x_{1}+3{x_{1}}^{2}-14x_{2}+6x_{1}x_{2}+3{x_{2}}^{2}\right)\right]\;\;\\ \times \left[30+{\left(2x_{1}-3x_{2}\right)}^{2}\left(18-32x_{1}+12{x_{1}}^{2}+48x_{2}-36x_{1}x_{2}+27{x_{2}}^{2}\right)\right] \end{array}$	2	[−2,2]	3
	$F_{16}\left(x\right)=-\sum_{i=1}^{4}c_{i}\exp\left(-\sum_{j=1}^{6}a_{ij}{\left(x_{j}-p_{ij}\right)}^{2}\right)$	6	[0, 1]	−3.32
	$F_{17}\left(x\right)=-\sum_{i=1}^{5}{\left[\left(X-a_{i}\right){\left(X-a_{i}\right)}^{T}+c_{i}\right]}^{-1}$	4	[0, 10]	−10.1532
	$F_{18}\left(x\right)=-\sum_{i=1}^{7}{\left[\left(X-a_{i}\right){\left(X-a_{i}\right)}^{T}+c_{i}\right]}^{-1}$	4	[0, 10]	−10.4028

**Table 3:** Optimization results of test functions (*N* = 30).

Test Function	Indicator	ICSA	CSA	DBO	SABO	GTO	ACO
$F_{1}\left(x\right)$	**Avg**	**0**	3.92351 × 10^−37^	3.5695 × 10^−211^	**0**	**0**	8.28236 × 10^−53^
	**Std**	**0**	8.74709 × 10^−37^	**0**	**0**	**0**	2.17691 × 10^−52^
	**Best**	**0**	1.53927 × 10^−39^	1.9754 × 10^−304^	**0**	**0**	3.08683 × 10^−56^
	**Worst**	**0**	4.05902 × 10^−36^	1.0708 × 10^−209^	**0**	**0**	1.04369 × 10^−51^
	**Ranking**	**1**	**4**	**2**	**1**	**1**	**3**
$F_{2}\left(x\right)$	**Avg**	1.3023 × 10^−256^	0.00075146	2.436 × 10^−110^	1.4396 × 10^−219^	**0**	9.08987 × 10^−32^
	**Std**	**0**	0.003940346	1.3342 × 10^−109^	**0**	**0**	9.92541 × 10^−32^
	**Best**	1.7608 × 10^−277^	3.57338 × 10^−16^	2.0974 × 10^−161^	2.3782 × 10^−223^	**0**	6.93638 × 10^−33^
	**Worst**	3.9048 × 10^−255^	0.021603366	7.308 × 10^−109^	2.1157 × 10^−218^	**0**	5.00026 × 10^−31^
	**Ranking**	**2**	**6**	**4**	**3**	**1**	**5**
$F_{3}\left(x\right)$	**Avg**	**0**	7.92906 × 10^−26^	9.4217 × 10^−181^	6.8492 × 10^−184^	**0**	2.56123 × 10^−08^
	**Std**	**0**	3.36937 × 10^−25^	**0**	**0**	**0**	5.22913 × 10^−08^
	**Best**	**0**	6.50681 × 10^−31^	1.6863 × 10^−296^	3.6308 × 10^−237^	**0**	1.9426 × 10^−10^
	**Worst**	**0**	1.80752 × 10^−24^	2.8265 × 10^−179^	2.0547 × 10^−182^	**0**	2.60581 × 10^−07^
	**Ranking**	**1**	**4**	**3**	**2**	**1**	**5**
$F_{4}\left(x\right)$	**Avg**	2.8539 × 10^−259^	0.099937236	2.7047 × 10^−103^	1.9581 × 10^−170^	**0**	4.76818 × 10^−14^
	**Std**	**0**	0.294700829	1.4814 × 10^−102^	**0**	**0**	1.39493 × 10^−13^
	**Best**	4.715 × 10^−280^	3.21841 × 10^−10^	5.5798 × 10^−160^	3.0862 × 10^−174^	**0**	1.84925 × 10^−15^
	**Worst**	8.5334 × 10^−258^	1.415456126	8.1139 × 10^−102^	3.1922 × 10^−169^	**0**	7.76042 × 10^−13^
	**Ranking**	**2**	**6**	**4**	**3**	**1**	**5**
$F_{5}\left(x\right)$	**Avg**	0.011126134	12.67625006	4.119694993	7.273422533	**0.005978363**	3.593146727
	**Std**	0.028595984	25.74275684	0.280024448	0.585405957	**0.02622753**	0.963212705
	**Best**	5.46113 × 10^−11^	6.341773794	3.517200359	6.26547137	**2.02995 × 10^−11^**	0.005562942
	**Worst**	**0.120001233**	148.9390102	4.797145153	8.706996661	0.142492067	4.118201494
	**Ranking**	**2**	**6**	**4**	**5**	**1**	**3**
$F_{6}\left(x\right)$	**Avg**	**0**	**0**	7.60789 × 10^−30^	0.044641887	1.11302 × 10^−26^	**0**
	**Std**	**0**	**0**	1.96701 × 10^−29^	0.08678659	3.01425 × 10^−26^	**0**
	**Best**	**0**	**0**	3.08149 × 10^−33^	0.000247241	4.93038 × 10^−32^	**0**
	**Worst**	**0**	**0**	8.81829 × 10^−29^	0.267369484	1.22077 × 10^−25^	**0**
	**Ranking**	**1**	**1**	**2**	**4**	**3**	**1**
$F_{7}\left(x\right)$	**Avg**	**0**	6.898379889	1.227114827	**0**	**0**	30.17059974
	**Std**	**0**	3.314695864	4.758636727	**0**	**0**	4.688056919
	**Best**	**0**	1.989918128	**0**	**0**	**0**	17.87799085
	**Worst**	**0**	16.91429035	21.88907407	**0**	**0**	38.05089992
	**Ranking**	**1**	**3**	**2**	**1**	**1**	**4**
$F_{8}\left(x\right)$	**Avg**	**8.88178 × 10^−16^**	8.853789839	**8.88178 × 10^−16^**	4.20404 × 10^−15^	**8.88178 × 10^−16^**	4.44089 × 10^−15^
	**Std**	**0**	9.865134386	**0**	9.01352 × 10^−16^	**0**	**0**
	**Best**	**8.88178 × 10^−16^**	4.44089 × 10^−15^	**8.88178 × 10^−16^**	8.88178 × 10^−16^	**8.88178 × 10^−16^**	4.44089 × 10^−15^
	**Worst**	**8.88178 × 10^−16^**	19.96610689	**8.88178 × 10^−16^**	4.44089 × 10^−15^	**8.88178 × 10^−16^**	4.44089 × 10^−15^
	**Ranking**	**1**	**4**	**1**	**2**	**1**	**3**
$F_{9}\left(x\right)$	**Avg**	**0**	0.15576281	0.015919282	**0**	**0**	0.366971328
	**Std**	**0**	0.091722932	0.048064635	**0**	**0**	0.133866692
	**Best**	**0**	0.02709365	**0**	**0**	**0**	0.160998083
	**Worst**	**0**	0.408678261	0.219083352	**0**	**0**	0.68081313
	**Ranking**	**1**	**3**	**2**	**1**	**1**	**4**
$F_{10}\left(x\right)$	**Avg**	**4.71163 × 10^−32^**	9.45344 × 10^−07^	4.51664 × 10^−19^	0.028353224	2.75368 × 10^−26^	4.71486 × 10^−32^
	**Std**	**1.67022 × 10^−47^**	5.17786 × 10^−06^	2.39465 × 10^−18^	0.032799116	1.39317 × 10^−25^	1.76746 × 10^−34^
	**Best**	**4.71163 × 10^−32^**	1.62026 × 10^−26^	4.83264 × 10^−32^	0.000799525	5.00206 × 10^−32^	**4.71163 × 10^−32^**
	**Worst**	**4.71163 × 10^−32^**	2.83603 × 10^−05^	1.3124 × 10^−17^	0.174522618	7.6356 × 10^−25^	4.80844 × 10^−32^
	**Ranking**	**1**	**5**	**4**	**6**	**3**	**2**
$F_{11}\left(x\right)$	**Avg**	**1.34978 × 10^−32^**	5.42618 × 10^−05^	0.028077621	0.136136058	0.001717404	1.98295 × 10^−32^
	**Std**	**5.5674 × 10^−48^**	0.000155233	0.054185142	0.133885771	0.0076006	3.468 × 10^−32^
	**Best**	**1.34978 × 10^−32^**	2.86319 × 10^−29^	1.5963 × 10^−32^	0.001968216	2.95216 × 10^−32^	**1.34978 × 10^−32^**
	**Worst**	**1.34978 × 10^−32^**	0.00061552	0.196253808	0.421268851	0.040534756	2.03448 × 10^−31^
	**Ranking**	**1**	**3**	**5**	**6**	**4**	**2**
$F_{12}\left(x\right)$	**Avg**	**0.998003838**	**0.998003838**	1.294638635	4.338341963	**0.998003838**	1.031138073
	**Std**	**0**	4.12326 × 10^−17^	1.009382635	3.602957648	**0**	0.181483682
	**Best**	0.998003838	0.998003838	0.998003838	**0.999621813**	0.998003838	0.998003838
	**Worst**	**0.998003838**	**0.998003838**	5.928845125	13.21815788	**0.998003838**	1.9920309
	**Ranking**	**1**	**1**	**3**	**4**	**1**	**2**
$F_{13}\left(x\right)$	**Avg**	**0.000307486**	0.000743635	0.000555178	0.000841977	0.000338009	0.001110708
	**Std**	**1.9311 × 10^−19^**	0.000254304	0.000262639	0.001024763	0.000167181	0.000280454
	**Best**	**0.000307486**	0.000307517	**0.000307486**	0.000314297	**0.000307486**	0.000827985
	**Worst**	**0.000307486**	0.001223278	0.001248167	0.00482856	0.001223173	0.001873158
	**Ranking**	**1**	**4**	**3**	**5**	**2**	**6**
$F_{14}\left(x\right)$	**Avg**	**0.397887358**	**0.397887358**	**0.397887358**	0.486377268	**0.397887358**	**0.397887358**
	**Std**	**0**	2.17226 × 10^−15^	**0**	0.157585427	**0**	**0**
	**Best**	0.397887358	0.397887358	0.397887358	**0.397917333**	0.397887358	0.397887358
	**Worst**	**0.397887358**	**0.397887358**	**0.397887358**	1.03595475	0.397887358	**0.397887358**
	**Ranking**	**1**	**1**	**1**	**2**	**1**	**1**
$F_{15}\left(x\right)$	**Avg**	**3**	**3**	**3**	5.105701141	**3**	**3**
	**Std**	1.91632 × 10^−15^	1.1057 × 10^−13^	1.91455 × 10^−15^	5.170560766	2.13615 × 10^−15^	**1.35253 × 10^−15^**
	**Best**	**3**	**3**	**3**	3.000697943	**3**	**3**
	**Worst**	**3**	**3**	**3**	28.97158012	**3**	**3**
	**Ranking**	**1**	**1**	**1**	**2**	**1**	**1**
$F_{16}\left(x\right)$	**Avg**	**−3.310105859**	−3.278107229	−3.210739931	−3.288603505	−3.274437923	−3.278401027
	**Std**	**0.036277689**	0.058672177	0.091077317	0.049810669	0.059241218	0.058273385
	**Best**	−3.321995172	−3.321995172	−3.321995172	**−3.321736945**	−3.321995172	−3.321995172
	**Worst**	**−3.20310205**	−3.19804659	−3.020699112	−3.10979718	**−3.20310205**	**−3.20310205**
	**Ranking**	**1**	**4**	**6**	**2**	**5**	**3**
$F_{17}\left(x\right)$	**Avg**	**−10.15319968**	−9.311128423	−6.766641721	−4.943759086	**−10.15319968**	−5.772228226
	**Std**	6.73596 × 10^−15^	1.915117716	2.435375689	0.354830429	**6.50588 × 10^−15^**	3.651680149
	**Best**	**−10.15319968**	**−10.15319968**	**−10.15319968**	−5.054837209	**−10.15319968**	**−10.15319968**
	**Worst**	**−10.15319968**	−5.10077214	−4.851276173	−3.2791667	**−10.15319968**	−2.630471668
	**Ranking**	**1**	**2**	**3**	**5**	**1**	**4**
$F_{18}\left(x\right)$	**Avg**	**−10.40294057**	−10.04996102	−7.81624257	−5.00084003	**−10.40294057**	−8.639825919
	**Std**	**8.07992 × 10^−16^**	1.343317601	2.847195896	0.518837042	9.32988 × 10^−16^	2.991783299
	**Best**	**−10.40294057**	**−10.40294057**	**−10.40294057**	−6.509305951	**−10.40294057**	**−10.40294057**
	**Worst**	**−10.40294057**	−5.087671825	−2.765897328	−3.224668878	**−10.40294057**	−2.765897328
	**Ranking**	**1**	**2**	**4**	**5**	**1**	**3**
**Average Ranking**		**1.17**	**3.33**	**3**	**3.28**	**1.67**	**3.17**
**Final Ranking**		**1**	**6**	**3**	**5**	**2**	**4**

**Table 4:** The mountain peaks information in terrain 1 and terrain 2.

Terrain	Mountain Peak	Center Coordinate	Height	The Descending Slope of the Peaks Along the x-Axis	The Descending Slope of the Peaks Along the y-Axis
Terrain 1	Peak 1	[300, 300]	55	90	90
	Peak 2	[500, 500]	60	105	105
	Peak 3	[800, 800]	80	60	60
	Peak 4	[250, 700]	70	60	60
	Peak 5	[750, 225]	65	75	75
	Peak 6	[550, 750]	55	90	90
	Peak 7	[850, 600]	75	60	60
Terrain 2	Peak 1	[750, 250]	55	90	90
	Peak 2	[750, 600]	75	120	120
	Peak 3	[250, 450]	80	75	75
	Peak 4	[450, 800]	70	90	90

**Table 5:** The cylindrical obstacle threat area information in Terrain 1 and Terrain 2.

Terrain	Obstacle Threat Area	The Center Coordinates of the Threat Area	The Radius of the Threat Area
Terrain 1	Threat 1	[250, 700]	150
Terrain 2	Threat 2	[500, 500]	150

**Table 6:** Path-planning results in different terrains.

Terrain	Indicator	ICSA	CSA	DBO	SABO	GTO	ACO
Terrain 1	**Avg**	**809.1455**	837.2897	835.7821	932.447	809.3106	827.3973
	**Std**	**8.2277**	19.4397	29.1549	170.8612	9.2895	10.8471
	**Best**	793.578	816.3347	794.6715	835.8318	**793.4277**	798.1731
	**Worst**	**816.6946**	876.5999	896.3591	1536.7116	819.239	855.612
	**Ranking**	**1**	**5**	**4**	**6**	**2**	**3**
Terrain 2	**Avg**	**819.0577**	824.8078	845.8159	1053.9564	824.4381	831.7481
	**Std**	**1.2546**	3.8733	18.6131	151.9352	14.1315	20.9946
	**Best**	818.1939	818.2718	818.4718	944.4681	**818.1781**	818.5506
	**Worst**	**821.6343**	833.3729	886.5101	1655.4466	865.5335	867.3063
	**Ranking**	**1**	**3**	**5**	**6**	**2**	**4**
**Average Ranking**		**1**	**4**	**4.5**	**6**	**2**	**3.5**
**Final Ranking**		**1**	**4**	**5**	**6**	**2**	**3**

## Data Availability

The data that support the findings of this study are available from the corresponding author upon request. There are no restrictions on data availability.
